# The Hedgehog–GLI1 Pathway Regulates Osteogenic Differentiation of Human Cervical Posterior Longitudinal Ligament Cells by BMP Signalling Pathway

**DOI:** 10.1111/jcmm.70393

**Published:** 2025-02-05

**Authors:** Wenbo Xu, Bingbing Ran, Toshimi Aizawa, Wanguo Liu, Jianhui Zhao, Renrui Niu, Zeping Liu, Rui Gu

**Affiliations:** ^1^ Department of Orthopaedic Surgery China‐Japan Union Hospital of Jilin University Jilin People's Republic of China; ^2^ Department of Ultrasound The First Hospital of Jilin University Jilin People's Republic of China; ^3^ Department of Orthopaedic Surgery Tohoku University School of Medicine Sendai Japan

**Keywords:** BMP2, BMP4, GLI1, Hedgehog signalling, ossification of posterior longitudinal ligament

## Abstract

Cervical ossification of the posterior longitudinal ligament (OPLL) is an ectopic ossification disorder characterised by endochondral ossification. Its aetiology remains to be fully elucidated. This study aimed to clarify its pathogenesis through RNA sequencing of primary cells cultured from patients without cervical OPLL (control, PLL) and patients with cervical OPLL (disease, OPLL). We revealed for the first time the role of GLI1 within OPLL cells. Functional experiments indicated that GLI1, acting as a pivotal mediator between the upstream Hedgehog pathway and downstream BMP pathway, influences the pathogenesis of OPLL. The positive/negative effects on osteogenic differentiation following activation/inhibition of the Hedgehog pathway can be rescued by manipulating GLI1 expression. Overexpression of GLI1 activates BMP signalling, enhancing osteogenic capacity in PLL cells, while GLI1 knockdown suppresses BMP signal transduction, attenuating osteogenic differentiation in OPLL cells. Our findings highlight the significant role of the canonical Hedgehog signalling pathway and its interaction with the BMP pathway in the pathogenesis of OPLL.

## 1 Introduction

Ossification of the posterior longitudinal ligament (OPLL) is a degenerative disease where ligament tissue is replaced by ectopic bone (bone forming in abnormal locations), predominantly affecting the cervical spine [1]. Severe cases can lead to compression of the spinal cord and nerve roots, resulting in various degrees of myelopathy and/or radiculopathy, including sensory and motor impairments, alongside sphincter dysfunction [2]. Notably, OPLL can render patients susceptible to mild traumas with potentially severe consequences, such as paralysis, imposing significant burdens on families and society [3]. Currently, surgical intervention remains the sole treatment approach for symptomatic OPLL, despite its inherent complexity and substantial risks, potentially leading to severe complications [4]. Nevertheless, surgery alone cannot halt the ongoing ossification and continuous progression of OPLL [5].

Despite extensive research and the identification of significant causal factors such as type 2 diabetes mellitus, increased body mass index and high bone mineral density influencing OPLL [6], the underlying mechanisms triggering OPLL remain elusive. Both nongenetic and genetic factors contribute to the disease progression, with external mechanical stresses [7] and internal cellular cytokines and growth factors [8, 9] exerting influences on ossification process. Some of these findings have already been validated in animal models of OPLL [10, 11]. It suggests that gaining insights into the regulatory mechanisms governing ectopic ossification in OPLL may offer promising avenues to impede disease progression and offer therapeutic benefits.

GLI Family Zinc Finger 1 (GLI1) is a transcription factor that is a part of the Hedgehog (Hh) signalling pathway, exerting crucial functions in embryonic development, tissue homeostasis and cellular proliferation across diverse tissues and organs [12]. The activation of GLI family transcription factors is facilitated by the binding of Sonic Hedgehog (Shh) to Patched‐1 (PTCH1), leading to the accumulation of GLI1 in the nucleus and subsequently influencing the expression of downstream target genes [13]. Despite research confirming the involvement of Hh pathways in intramembranous and endochondral ossification [14], the debate on whether it functions as a positive or negative regulatory factor in this process persists [15, 16, 17, 18]. In this study, we utilised transcriptome analysis to identify differential expression of GLI1 in OPLL and PLL, and established its role as a potent enhancer of osteogenesis in OPLL cells. Further investigations uncovered a potential mechanism in which BMP pathway, acting as a crucial intermediary, bridges the influence of GLI1 on OPLL occurrence. Understanding this relationship could offer new avenues for therapeutic intervention in OPLL with further validation.

## 2 Materials and Methods

### 2.1 Sample Collection and Process

The clinical characteristics of the patients are presented in Table S1. Diagnosis of OPLL or non‐OPLL was confirmed preoperatively through computed tomography (CT) and magnetic resonance imaging (MRI). Exclusion criteria encompassed: (1) Trauma‐related cases involving cervical spine fractures or spinal cord injuries; (2) Severe osteoporosis, metabolic bone disorders, spinal neoplasms, or spinal infections as concurrent conditions.

In terms of sample processing, the posterior longitudinal ligament (PLL) tissues obtained from anterior cervical surgery were thoroughly cleansed and promptly utilised for primary cell culture or immunohistochemistry.

Precisely, tissue blocks of approximately 1 mm^3^ were trimmed and situated within precoated culture dishes containing 0.1% gelatine, subsequently filled with complete medium (90% high‐glucose Dulbecco's modified Eagle's medium (DMEM, HyClone), 10% foetal bovine serum (FBS, VivaCell Biosciences) and 1% penicillin/streptomycin (HyClone)). These tissue blocks were then incubated at 37°C in a 5% CO_2_ humidified atmosphere. Within 14–30 days, a substantial population of fibroblast‐like cells migrated from the tissue and adhered to the dish surface. Upon reaching 80% confluence, cells were subcultured at a ratio of 1:3. Subsequent experiments were conducted using cells derived from passages 2–6.

### 2.2 Cell Characterisation

To substantiate the identity of the harvested cells, characterisation of surface marker expression and the assessment of their multipotent differentiation potential were conducted. Succinctly put, the identification of surface antigens on mesenchymal stem cells was performed using flow cytometry. Cells derived from both OPLL and non‐OPLL sources underwent enzymatic digestion using trypsin–EDTA (0.25%) (HyClone). Followed by thorough washing, cells were incubated with specific antibodies at room temperature in the absence of light for 15 min. Cells were analysed by a flow cytometer (BD Biosciences). Antibodies against human phycoerythrin (PE)‐conjugated CD34, PE‐conjugated CD45, allophycocyanin (APC)‐CD105, APC‐CD73, fluorescein isothiocyanate (FITC)‐conjugated CD 90 and FITC‐conjugated HLA‐DR were used in the study (all from TBD Science). For the purpose of clarity within this study, we termed primary cells derived from non‐OPLL and OPLL patients as PLL and OPLL cells respectively.

Further, we induced primary cells into osteogenic, adipogenic and chondrogenic differentiations. For osteogenic differentiation, primary cells were seeded in cell culture dishes at a density of 1 × 10^4^ cells/cm^2^. Until cells reached 70% confluence, the complete medium was substituted with osteogenic induction medium (DMEM supplemented with 10% FBS, 1% penicillin/streptomycin, 100 nM dexamethasone (Sigma‐Aldrich), 50 μg/mL ascorbic acid (Sigma‐Aldrich) and 10 mM β‐glycerophosphate (Sigma‐Aldrich)). Cells were maintained for 7–21 days. Adipogenic differentiation was initiated after the cells reached 100% confluence by alternating treatments with solution A and solution B (Cyagen Biosciences) for 21 days, as per the manufacturer's instructions. Similarly, chondrogenic differentiation was induced using Cyagen's chondrogenic medium for 14 days, in strict accordance with the manufacturer's protocol.

### 2.3 ALP Staining

ALP staining was conducted to evaluate osteogenic differentiation. Following osteogenic induction for 0–21 days, cells were rinsed with PBS and subsequently fixed using 4% paraformaldehyde. Cells were stained employing the BCIP/NBT Alkaline Phosphatase Colour Development Kit (Beyotime) in accordance with the manufacturer's instructions. Six fields of micrographs were randomly obtained and analysed for each sample. The measurement of the stained area was performed using ImageJ software.

### 2.4 Alizarin Red S Staining

Mineral deposition was evaluated by alizarin red S (ARS) staining. Cells were fixed with 95% ethanol for 10 min and then incubated with ARS (2%, pH 4.2) (Beyotime) at 37°C for 30 min. Meticulous sample washing and subsequent photography were undertaken. The degree of mineralisation was quantified by treatment with 10% cetylpyridinium chloride (CPC) monohydrate (Sigma‐Aldrich). A volume of 200 μL of the supernatant was taken from the above solution and placed in a 96‐well plate to measure absorbance at 562 nm.

### 2.5 Oil Red O Staining

To assess adipogenic differentiation, cells were fixed in 4% paraformaldehyde for 10 min and then incubated with 0.3% oil red O (Beyotime) at room temperature for 30 min. After extensive washing, cells were moistened with PBS, and images were captured using a light microscope (Olympus).

### 2.6 Toluidine Blue Staining

Toluidine Blue staining solution was used to assess chondrogenic differentiation. After 14 days of chondrogenic differentiation, toluidine blue was added to each well and incubated for 5 min according to the manufacturer's instructions. An equal amount of distilled water was then added and mixed, followed by a 15‐min incubation. After washing with distilled water, images were captured using a light microscope (Olympus).

### 2.7 Cell Apoptosis Assay

Detection of apoptosis was performed using the Annexin V‐FITC/PI apoptosis detection kit (BD Biosciences). Briefly, according to the manufacturer's protocol, 1 × 10^5^ cells were resuspended in 100 μL of the provided binding buffer and then stained with 5 μL of FITC‐conjugated Annexin V and 5 μL of PI in the dark at room temperature for 15 min. The fluorescence intensities of the cells were measured using a flow cytometer (BD Biosciences). Annexin V‐FITC−/PI− cells were considered normal, Annexin V‐FITC+/PI− cells indicated early apoptosis, Annexin V‐FITC+/PI+ cells indicated late apoptosis and Annexin V‐FITC−/PI+ cells indicated necrosis.

### 2.8 Quantitative Real‐Time Polymerase Chain Reaction Analysis

Total RNA extraction was conducted using TransZol (TransGen) following the manufacturer's protocol. Subsequently, the extracted RNA underwent reverse transcription into cDNA using PrimeScript RT Master Mix (TaKaRa). Quantitative real‐time PCR (qPCR) was performed using TransStart Green qPCR SuperMix (TransGen) on a Real‐Time PCR Detection System (ABI 7500) according to the manufacturer's instructions. Primer details are provided in Table S2. The 2^−ΔΔCt^ method was employed to calculate the relative gene expression with expression levels normalised to GAPDH.

### 2.9 Western Blot Analysis

For protein extraction, cells in good growth condition were washed three times with precooled PBS, then lysed on ice for 30 min in 50–100 μL of RIPA lysis buffer containing 1% PMSF. After lysing, the mixture was centrifuged at 13,000 rpm for 10 min at 4°C. The supernatant was collected, and the protein concentration was determined using a BCA protein assay kit (Beyotime) following the manufacturer's instructions. The separation between nuclear and cytoplasmic proteins was performed using a nuclear and cytoplasmic protein extraction kit (Beyotime). Electrophoresis was performed on 8%–12% sodium dodecyl sulphate–polyacrylamide gel using an equivalent protein load of 20 μg, followed by transfer onto polyvinylidene difluoride membranes (Millipore). The membranes were blocked using 5% nonfat dried milk for 1 h at room temperature and then incubated with various primary antibodies (Table S3) at 4°C overnight. After thorough washing, HRP‐conjugated secondary antibody (ProteinTech) was applied at a 1:5000 dilution. Blots were visualised by ECL (Tanon).

### 2.10 Transfection of Lentivirus

Commercial lentiviral systems from Hanheng Bio and GenePharma were employed for achieving GLI1 overexpression and knockdown. The targeted GLI1 sequence in shRNA was GCAGTAAAGCCTTCAGCAATG. The shRNA with noncomplementary sequences served as a negative control. The GLI1 coding sequence was synthesised and cloned into the HBLV‐3xflag‐ZsGreen‐PURO vector for overexpression. The empty lentivirus vector served as a negative control. For lentiviral transduction, cells at approximately 50% density were infected with a multiplicity of infection of 200, supplemented with 2 μg/mL polybrene (Beyotime). The complete culture medium was introduced after 48 h. Western blot (WB) analyses and GFP expression under fluorescence microscopy were conducted to confirm the efficiency of overexpression or knockdown.

### 2.11 RNA‐Seq and Bioinformatics Analysis

Total RNA was extracted utilising TransZol (TransGen) as per the manufacturer's protocol. The transcriptome sequencing was conducted with the help of OE Biotech Co. Ltd. (Shanghai, China). DESeq2 package facilitated differential gene expression analysis, with differentially expressed genes (DEGs) identified by meeting q‐value < 0.05 and fold change > 2 or fold change < 0.5. Subsequently, DEGs underwent enrichment analysis for Gene Ontology (GO), Kyoto Encyclopaedia of Genes and Genomes (KEGG), Reactome and WikiPathways to identify significantly enriched terms. Protein–protein interaction (PPI) network was built and visualised by Search Tool for the Retrieval of Interacting Genes (STRING) online database (http://string‐db.org; version 12.0) and cytoscape (version 3.10.1). Hub genes of PPI network were identified by plugin CytoHubba based on the scores of Maximal Clique Centrality (MCC) algorithm [19]. The analyses and visualisation were carried out using R statistical software (version 4.2.2).

### 2.12 Coimmunoprecipitation (Co‐IP) Assays

We performed Co‐IP using the Immunoprecipitation Kit (Beyotime). In brief, cells were harvested and washed three times with cold PBS. The cell pellets were resuspended in CoIP lysis buffer containing Protease Inhibitor Cocktail. The cell suspensions were kept on ice and subjected to 20 oscillations on a vortex shaker with 2‐min intervals. Afterwards, the samples were centrifuged at 12,000 rpm for 15 min at 4 °C. The supernatant was collected and slowly rotated overnight at 4 °C with primary antibodies. Subsequently, 30 μL of BeyoMag Protein A + G Magnetic Beads was added for 3 h. The beads were washed three times with 1 mL of CoIP lysis buffer containing protease inhibitor cocktail for 5 min each. The beads were then suspended in 100 μL 1× loading buffer and heated at 100 °C for 8 min. After removing the beads, the samples were analysed using WB.

### 2.13 Immunofluorescence Staining

Immunofluorescence was conducted on PLL and OPLL cells at different osteogenic induction time points (0, 7, 14 days) following this protocol: cells were fixed with 4% paraformaldehyde for 30 min and permeabilised with 0.2% Triton X‐100 for 15 min. To assess GLI1 nuclear translocation, cells were blocked with 5% bovine serum albumin (BSA) for 60 min and then incubated with a mouse monoclonal anti‐GLI1 antibody overnight at 4°C. After thorough rinsing, cells were incubated with anti‐mouse IgG (H + L) (Alexa Fluor 488 Conjugate, Cell Signalling Technology) for 2 h. Nuclei were counterstained with DAPI for 10 min followed by image capture using an Olympus FV‐1000 confocal laser scanning microscope. The ratio of nucleus versus cytoplasm was calculated by ImageJ software.

### 2.14 Immunohistochemistry

To compare GLI1 expression between PLL and OPLL, immunohistochemistry staining was conducted on tissue sections. Following fixation in 10% neutral buffered formalin at room temperature for 24 h, the PLL tissues were dehydrated and embedded in paraffin. Sections (5 μm thick) were then cut and underwent standard deparaffinisation and rehydration. Antigen retrieval was performed, followed by blocking to minimise nonspecific binding. Primary antibodies were applied overnight at 4°C. After washing, sections were incubated with secondary antibodies for 30 min at 37°C. Visualisation utilised a diaminobenzidine (DAB) substrate, with haematoxylin counterstaining for nuclei. The positive signal was brown. Stained sections were examined under a light microscope (Olympus). Positive staining average area percentage was quantified using the ImageJ software.

### 2.15 Cell Counting Kit‐8 (CCK‐8) Assay

To assess the proliferative capacity of PLL and OPLL cells, we employed the Cell Counting Kit‐8 (CCK‐8, Invigentech). A total of 1000 cells in 100 μL of medium were seeded into each well of a 96‐well plate. Following a 2‐h incubation, 10 μL of CCK‐8 reagent was added for staining. The absorbance was measured at 450 nm using spectrophotometry from day 1 to day 6. In addition, we employed the CCK‐8 assay to establish safe drug concentrations following the manufacturer's guidelines. Primary cells, with 3000 cells per well, were seeded into a 96‐well plate. After 24 h of incubation, we added Purmorphamine (PM) to PLL cells and Cyclopamine (CPN) to OPLL cells at appropriate concentration gradients. Subsequent to another 48‐h incubation, 10 μL of CCK‐8 reagent was introduced to each well and incubated for roughly 2 h. We gauged the absorbance at 450 nm using a microplate reader.

### 2.16 Statistical Analysis

Statistical analysis was performed by GraphPad Prism 9.5.0 software. All data are expressed as the mean ± SEM. Two‐tailed Student's t‐test was conducted to determine the significance between two independent variables. One‐way ANOVA was used to compare differences between multiple independent variables. *p*‐value < 0.05 was considered to be statistically significant.

## 3 Results

### 3.1 PLL and OPLL Cells Exhibit Phenotype of MSCs and Multipotent Differentiation Potential

**FIGURE 1:**
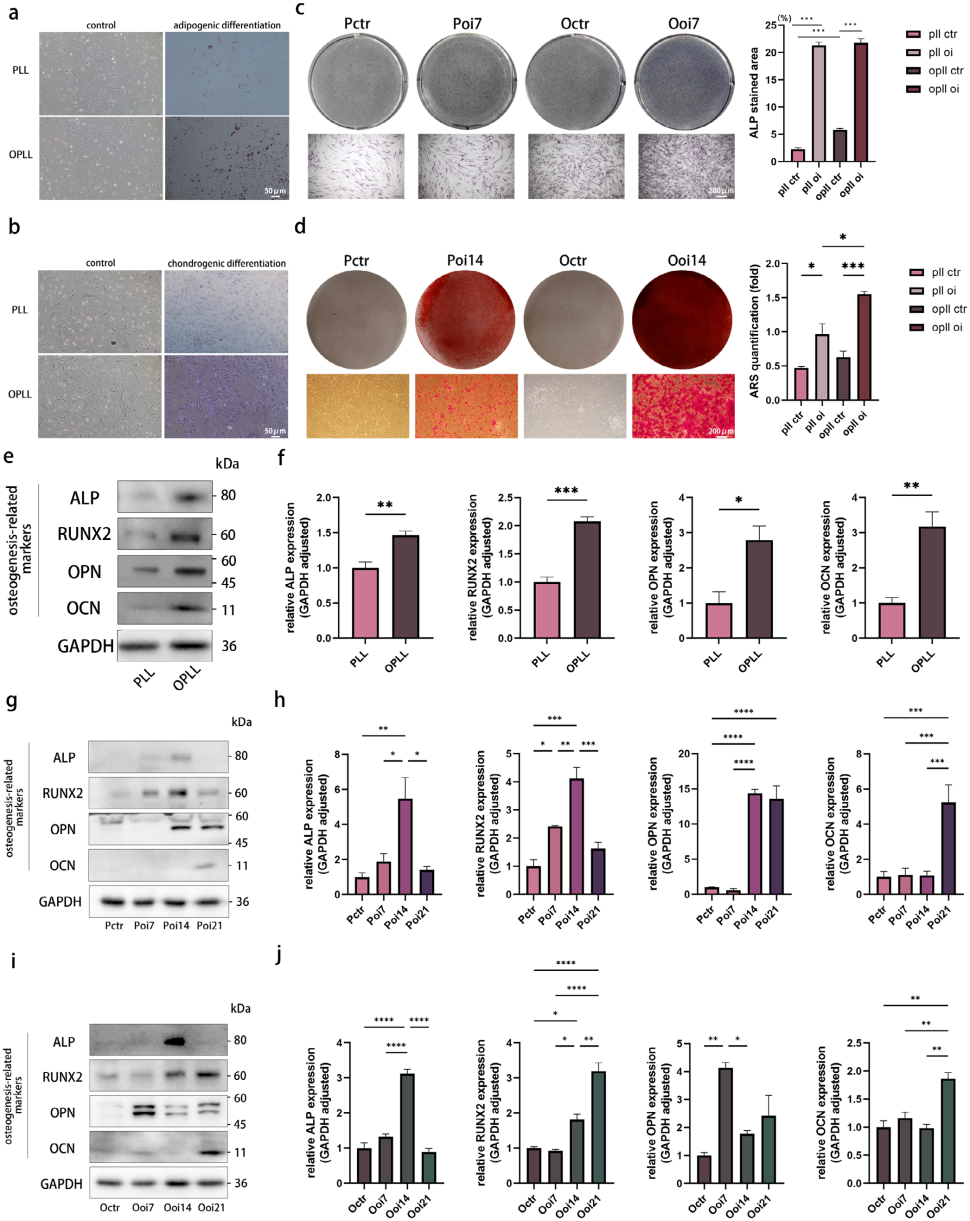
Culture and phenotypic characteristics of primary cells from control (PLL) and disease (OPLL) groups. (a) Adipogenic differentiation of primary cells from PLL and OPLL with or without adipogenic induction, stained with oil red O. (b) Chondrogenic differentiation of primary cells from PLL and OPLL with or without chondrogenic induction, stained with toluidine blue. Osteogenic differentiation was induced for 0–14 days in PLL and OPLL (Pctr, Poi7, Poi14, Octr, Ooi7 and Ooi14) using osteogenic induction medium, followed by (c) ALP and (d) Alizarin Red S (ARS) staining. ‘Pctr’ and ‘Octr’ represent uninduced cells, while ‘Poi7’, ‘Poi14’, ‘Ooi7’ and ‘Ooi14’ represent cells induced for 7 and 14 days respectively. (e, f) Evaluation of osteogenesis‐related markers in PLL and OPLL through western blot (WB) analysis, with statistical analysis. (g, h) Time‐dependent osteogenic differentiation in PLL cells, with protein expression levels of osteogenesis‐related markers measured at 0 (Pctr), 7 (Poi7), 14 (Poi14) and 21 (Poi21) days. (i, j) Time‐dependent osteogenic differentiation in OPLL cells, with protein expression levels of osteogenesis‐related markers measured at 0 (Octr), 7 (Ooi7), 14 (Ooi14) and 21 (Ooi21) days. *n* = 3 per group. Data presented as mean ± SEM. Significant levels are *p < 0.05, **p < 0.01, ***p < 0.001 and ****p < 0.0001. Scale bar: As shown in the figure.

Flow cytometry revealed the expression of typical MSC surface markers [20], CD105, CD73 and CD90, in both PLL and OPLL cells, while CD45, CD34 and HLA‐DR were negative (Figure S1a). Moreover, osteogenic, adipogenic and chondrogenic differentiation capabilities were shared by both cell types. Specifically, oil red O staining verified their ability for adipogenesis, albeit with moderate intensity (Figure 1a), while toluidine blue staining demonstrated their chondrogenic features (Figure 1b). ALP staining indicated higher activity in OPLL cells, which persisted after osteogenic differentiation (Figure 1c). Compared to PLL cells, OPLL cells exhibited enhanced mineralisation capacity, regardless of osteogenic differentiation (Figure 1d). Additionally, significantly elevated protein levels of early osteogenic markers like ALP [21] and RUNX2 and late mineralisation markers such as OPN and OCN were noted in OPLL cells [22] (Figure 1e,f). Furthermore, from day 2 to day 6, OPLL cells exhibited significantly higher proliferative capacity compared to PLL cells (Figure S1b). Under the same passage conditions, OPLL cells also showed a higher apoptosis rate (Figure S1c).

To gain deeper insights into the osteogenic traits of both two cell types, we performed a time‐dependent analysis of osteogenesis‐related markers through WB. We observed that the expression of osteogenesis‐related proteins exhibited a unimodal trend, wherein the protein expression gradually increased with prolonged osteogenic induction and then gradually decreased following a peak (Figure 1e–j). In the case of ALP, a statistically significant increase occurred between the 7th and 14th days of osteogenic induction, followed by a sharp decline. This phenomenon was consistent in both PLL and OPLL. In PLL, the expression of RUNX2 showed a gradual increase during osteogenic induction, but a change in this trend occurred on the 14th day, resulting in decreased expression levels. In OPLL, a statistically significant increase occurred after the 7th day of osteogenic induction and continued until the 21st day. There were significant differences in OPN expression between the two cell types. In OPLL, the peak expression of OPN was observed on the 7th day of osteogenic induction, while in PLL, OPN exhibited minimal alterations in the early stages of osteogenic induction but exhibited a sharp increase on the 14th day, which persisted until the 21st day. In alignment with the existing knowledge, the protein expression of OCN, a hallmark of late‐stage mineralisation in bone formation, exhibited a notable increase significantly on the 21st day of osteogenic induction. This trend remained uniform in both OPLL and PLL.

### 3.2 GLI1 is Highly Expressed in OPLL Cell Both In Vivo and In Vitro

**FIGURE 2:**
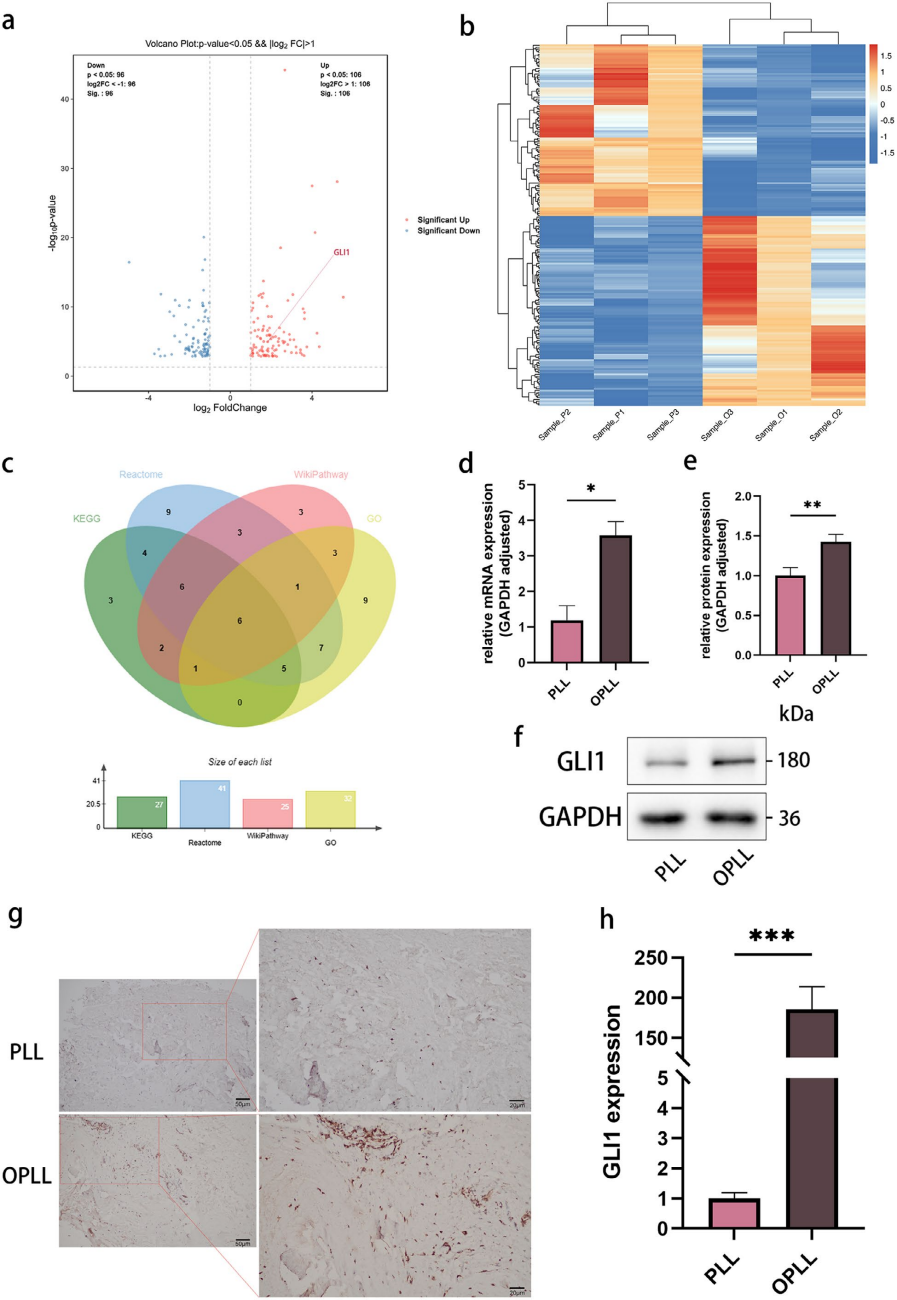
Identification and validation of key differentially expressed genes (DEGs) in OPLL and PLL. (a) Volcano plot illustrating differentially expressed mRNAs between OPLL and PLL. (b) Heatmap displaying the differential mRNA expression profile between OPLL and PLL. (c) Strategy for identifying key DEG. (d) Gene expression of GLI1 determined by qRT‐PCR. (e, f) Protein expression and quantitative analysis of key DEG (GLI1) assessed through WB. (g, h) Representative images and quantitative analysis of GLI1 immunohistochemistry (IHC) staining. *n* = 3 per group. Data presented as mean ± SEM. Significant levels are *p < 0.05, **p < 0.01 and ***p < 0.001. Scale bar: As shown in the figure.

Transcriptomic analysis provided us with a vivid depiction of DEGs landscape between PLL and OPLL cells (Figure 2a). Comparative analysis revealed a total of 233 DEGs, including 128 upregulated and 105 downregulated genes (Figure 2b). To elucidate the functional and biological properties of DEGs, we conducted GO analyses encompassing biological process, cellular component and molecular function. It identified 86 significantly associated GO terms (*p* < 0.01) with upregulated genes, including cartilage condensation, chondrocyte development, osteoblast differentiation and ossification (Figure S2). Employing three biological databases (KEGG, Reactome and WikiPathways), we conducted further pathway analyses of the DEGs. In the WikiPathways enrichment analysis, we noted a substantial enrichment of the endochondral ossification pathway among upregulated genes, while the osteoclast signalling pathway was enriched among downregulated genes (Figure S3). This observation aligns with previous literature on the pathogenesis of OPLL [23]. Subsequently, we sifted through DEGs associated with bone formation and ectopic bone formation processes within top 30 enrichment GO terms and took the intersection of these genes with the DEGs derived from the top 20 upregulated pathways across three databases, in an attempt to unveil key DEGs relevant to OPLL (Figure 2c). GLI1 emerged as a compelling candidate, emphasising both statistical and biological significance.

**FIGURE 3:**
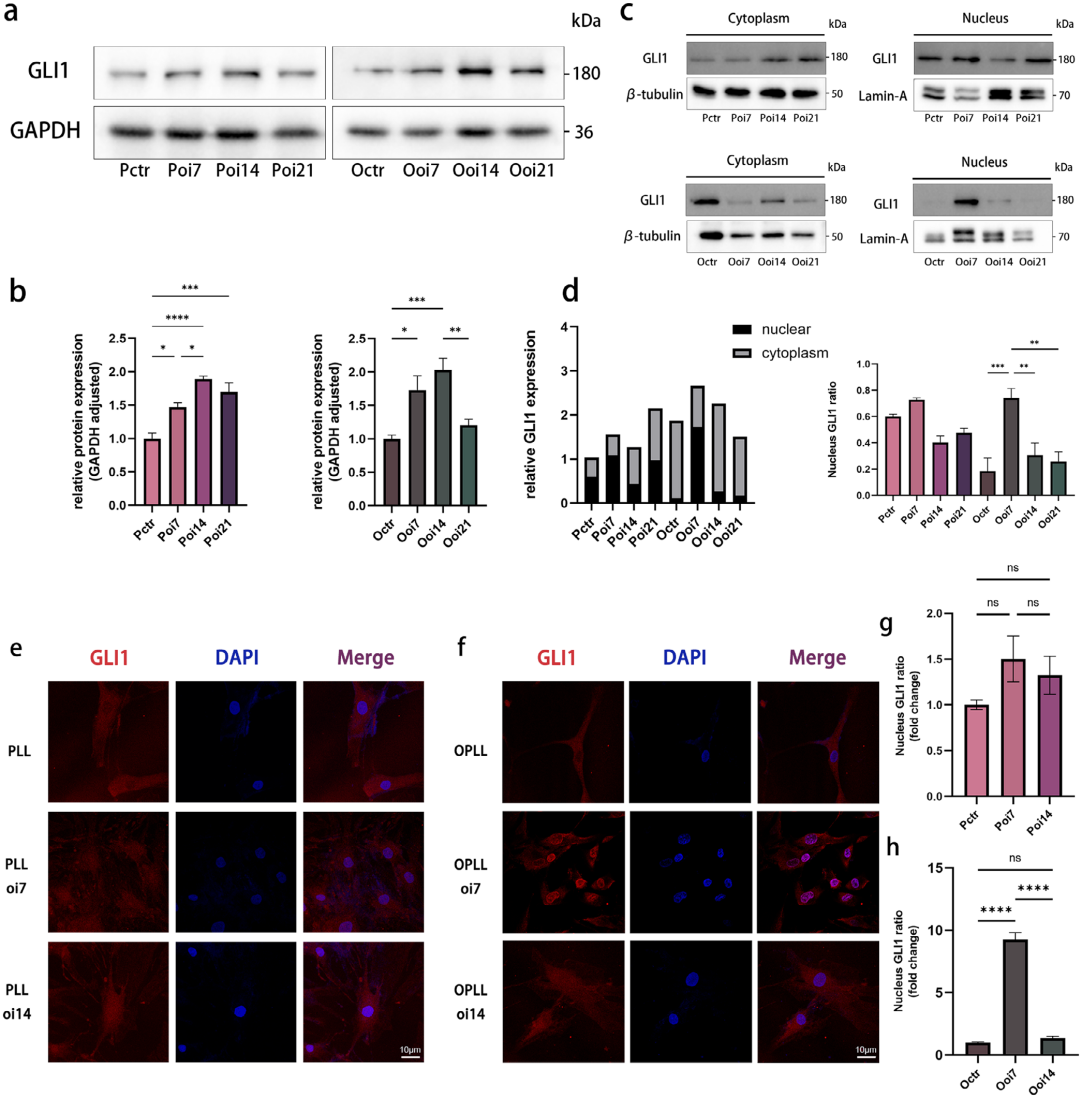
Dynamic changes of GLI1 during osteogenic differentiation in PLL and OPLL cells. (a, b) Protein expression levels of GLI1 measured at 0, 7, 14 and 21 days during time‐dependent osteogenic differentiation in PLL and OPLL cells, with quantitative analysis. (c, d) Protein levels of GLI1 in the cytoplasm and nucleus during time‐dependent osteogenic differentiation, accompanied by quantitative analysis of the nucleus GLI1 protein ratio. (e, f) Representative immunofluorescence staining images; red (GLI1) and blue (nuclei). (g, h) Quantitative analysis of the fluorescence intensity of GLI1. *n* = 3 per group. Data presented as mean ± SEM. Significant levels are *p < 0.05, **p < 0.01, ***p < 0.001 and ****p < 0.0001. ns: No significance. Scale bar: As shown in the figure.

We gauged the mRNA and protein expression levels of GLI1 in both cell types using qPCR and WB. In comparison to PLL cells, OPLL cells exhibited a significant increase in GLI1 expression at both the mRNA and protein levels (Figure 2d–f), aligning with the heightened in vivo GLI1 levels observed via IHC (Figure 2g,h). Subsequently, the protein expression levels of GLI1 were observed in both PLL and OPLL cells during different stages of osteogenic induction. Remarkably, a pattern of initial elevation followed by subsequent downregulation in GLI1 expression was observed, implying the early involvement of GLI1 in the OPLL disease progression (Figure 3a,b). Furthermore, we also found an enhanced nuclear translocation of GLI1 upon osteogenic differentiation induction (Figure 3c–h). Following the separation of nuclear and cytoplasmic proteins, we performed individual WB analyses on OPLL and PLL cells (Figure 3c). The results highlighted an intriguing disparity: during the initial phases of osteogenic differentiation, OPLL cells exhibited a significant nuclear translocation of GLI1, whereas the nuclear GLI1 content in PLL cells remained relatively stable. Quantitative analysis of relative grayscale and fluorescence intensity further substantiated these alterations (Figure 3d,g,h).

### 3.3 Hedgehog Pathway Regulates Osteogenic Differentiation of OPLL and PLL Cells via GLI1

**FIGURE 4:**
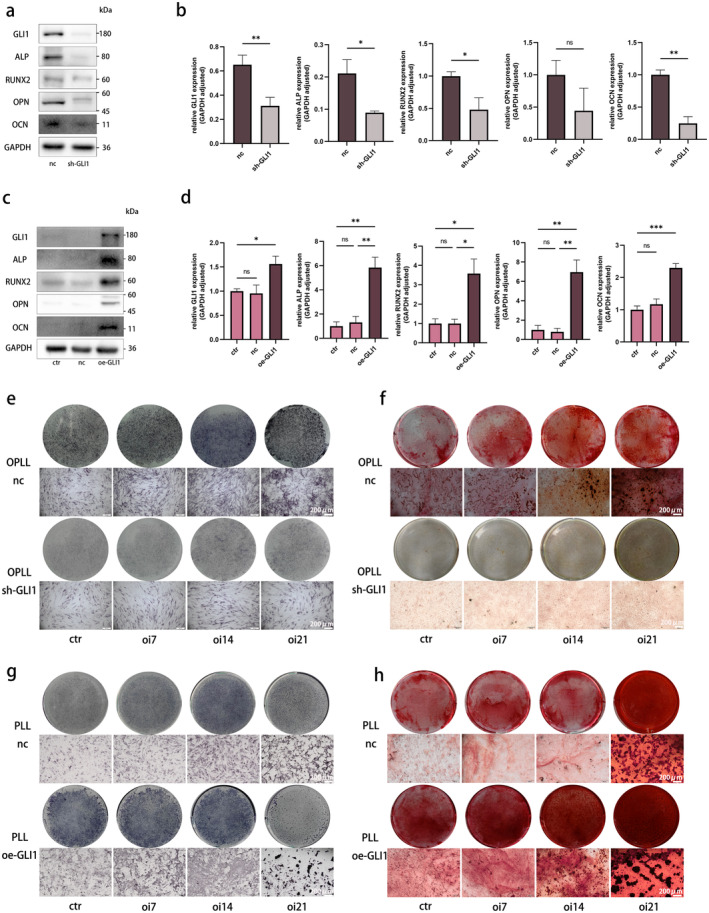
GLI1 facilitates osteogenic differentiation in PLL and OPLL cells. (a, b) Evaluation of the impact of GLI1 knockdown on osteogenesis‐related markers expression in OPLL cells through WB, with quantitative analysis. (c, d) Assessment of the influence of GLI1 overexpression on osteogenesis‐related markers expression in PLL cells through WB, with quantitative analysis. (e, f) Osteogenic differentiation in OPLL cells transfected with negative control and sh‐GLI1, followed by ALP staining and ARS staining at 0, 7, 14 and 21 days. (g, h) Osteogenic differentiation in PLL cells transfected with negative control and oe‐GLI1, followed by ALP staining and ARS staining at 0, 7, 14 and 21 days. *n* = 3 per group. Data presented as mean ± SEM. Significant levels are *p < 0.05, **p < 0.01 and ***p < 0.001. ns: No significance. Scale bar: As shown in the figure.

In order to further validate the function of GLI1 in OPLL, stable cell lines with GLI1 knockdown and overexpression were established in OPLL cells and PLL cells respectively (Figure 4a,c and Figure S4). Following GLI1 knockdown, there was a significant decrease in the protein levels of osteogenesis‐related markers (ALP, RUNX2, OPN and OCN), accompanied by diminished ALP secretion and mineralisation capacity in OPLL cells (Figure 4a,b,e,f). Conversely, GLI1 overexpression led to a substantial upregulation of these genes and an enhanced ALP‐secretion and mineralisation ability in PLL cells (Figure 4c,d,g,h).

**FIGURE 5:**
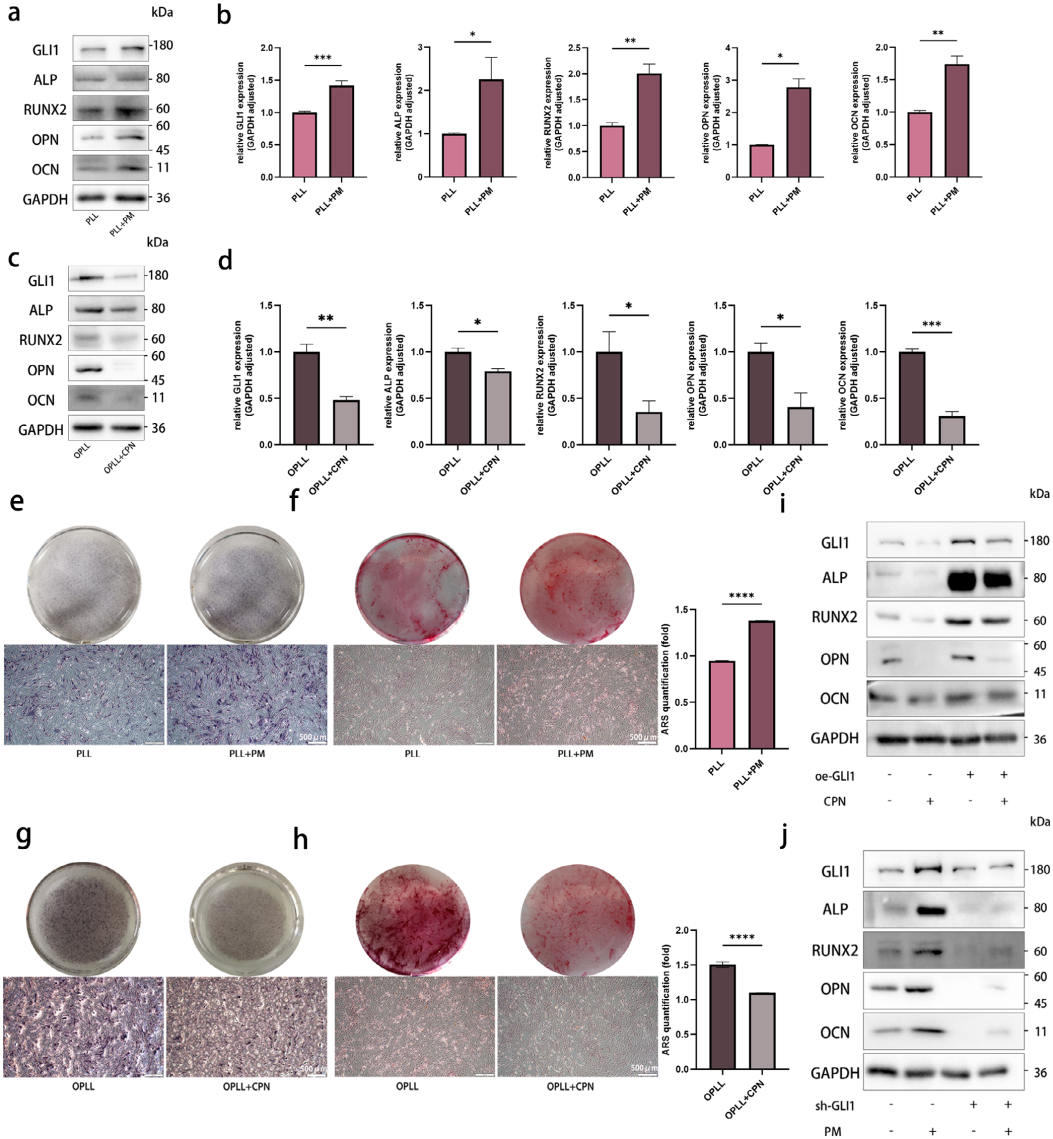
Hedgehog (Hh) pathway facilitates osteogenic differentiation in PLL and OPLL cells through GLI1. (a, b) Evaluation of the influence of DMSO (control) and PM on osteogenesis‐related markers expression in PLL cells through WB, with quantitative analysis. (c, d) Assessment of the impact of ethanol (control) and CPN on osteogenesis‐related markers expression in OPLL cells through WB, with quantitative analysis. (e, f) Osteogenic differentiation in PLL cells treated with DMSO (control) and PM, followed by ALP staining and ARS staining at 7 and 14 days, with absorbance measurement of dissolved ARS at 562 nm. (g, h) Osteogenic differentiation in OPLL cells treated with ethanol (control) and CPN, stained for ALP and ARS at 7 and 14 days, with absorbance measurement of dissolved ARS at 562 nm. (i) WB evaluation of GLI1 and osteogenesis‐related markers protein expression in PLL cells under three conditions: GLI1 overexpression, CPN addition and a combination of GLI1 overexpression with CPN supplementation. (j) WB assessment of GLI1 and osteogenesis‐related markers protein expression in OPLL cells under three conditions: GLI1 knockdown, PM addition and a combination of GLI1 knockdown with PM supplementation. *n* = 3 per group. Data presented as mean ± SEM. Significant levels are *p < 0.05, **p < 0.01, ***p < 0.001 and ****p < 0.0001. Scale bar: As depicted in the figure.

Building upon the pivotal role of GLI1 in the Hh pathway, we delved deeper to investigate whether the Hh pathway is implicated in the development of OPLL. Purmorphamine (PM) is a small molecule that selectively activates the Hh signalling pathway, leading to the activation of GLI1 [24]. CPN serves as an antagonist of Hh pathway, sharing the same target as PM [25]. We first identified the safe concentration for PM as 2 μM using the CCK8 method, while the corresponding value for CPN was 20 μM (Figure S5). Next, the addition of PM to PLL cells was found to be highly effective in inducing mineralisation. PM significantly elevated the expression levels of GLI1 and, concurrently, strongly induced aforementioned osteogenesis‐related markers (Figure 5a,b,e,f). In the case where CPN was introduced during OPLL cell culture, as envisaged, it led to a notable decrease in osteogenesis process (Figure 5c,d,g,h).

To further elucidate the mechanistic underpinnings, we selectively inhibited the Hh pathway while concurrently overexpressing GLI1. Our results revealed a restorative effect on osteogenesis‐related markers, strongly suggesting that GLI1 acts as a key mediator through which the Hh pathway exerts its regulatory influence on osteogenic differentiation (Figure 5i and Figure S6a). Conversely, the elimination of GLI1, even under Hh pathway activation, sustained osteogenesis‐related markers at diminished levels (Figure 5j and Figure S6b).

### 3.4 GLI1 Regulates OPLL Through BMP Pathway

**FIGURE 6:**
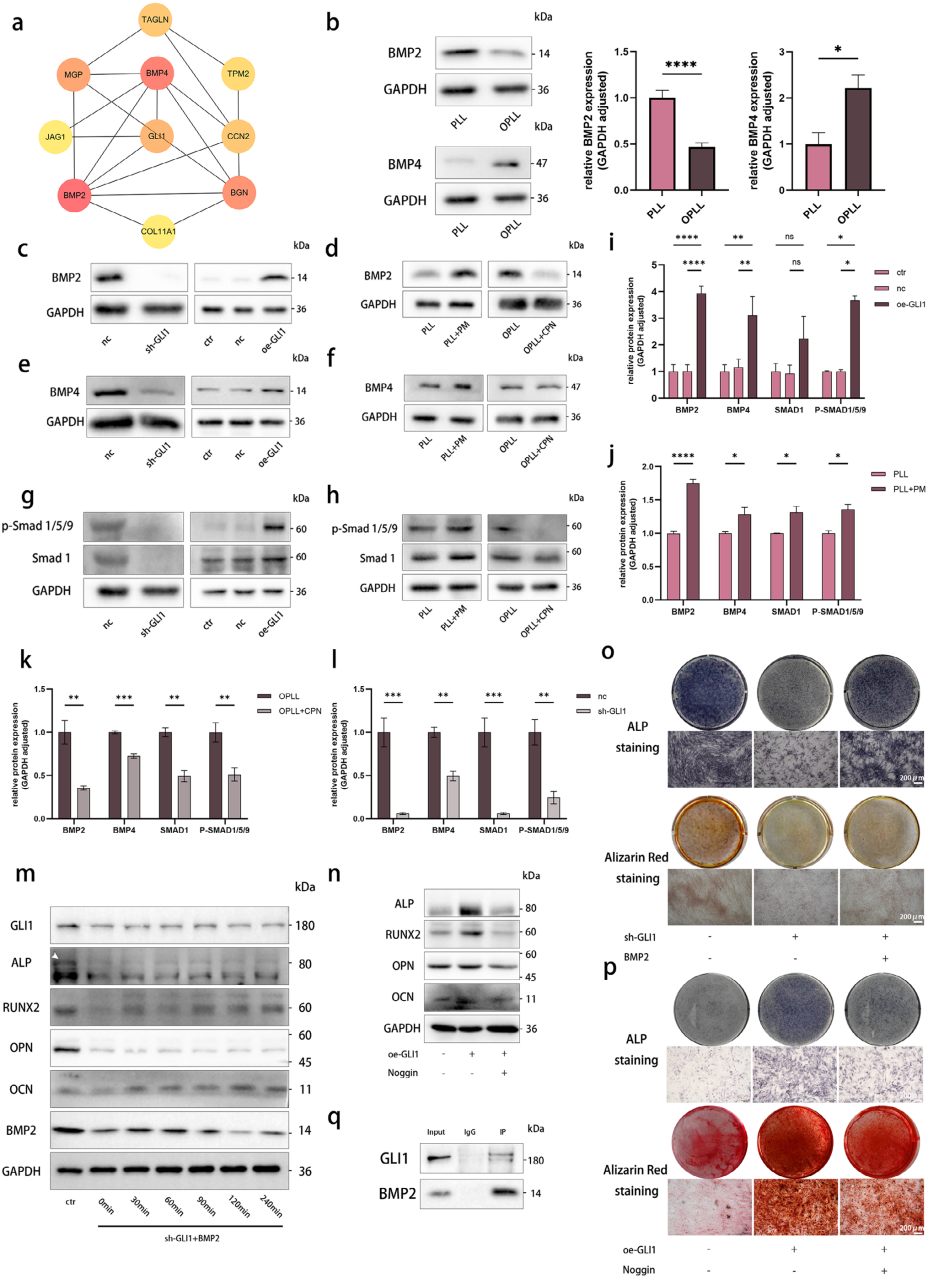
GLI1 regulate osteogenic differentiation in PLL and OPLL cells via the BMP signalling pathway. (a) Protein–protein interaction network highlighting hub genes. (b) Evaluation of BMP2 and BMP4 protein expression levels in PLL and OPLL through WB, with statistical analysis. (c, e, g, i, l) Evaluation of influence of GLI1 knockdown in OPLL cells and GLI1 overexpression in PLL cells on the protein expression levels of BMP2, BMP4, Smad1 and *p*‐Smad1/5/9 in the BMP pathway, assessed by WB and quantified. (d, f, h, j, k) Evaluation of the influence of DMSO (control) and PM in PLL cells, and ethanol (control) and CPN in OPLL cells, on the protein expression levels of BMP2, BMP4, Smad1 and *p*‐Smad1/5/9 in the BMP pathway, assessed by WB and quantified. (m) Protein expression of GLI1 and osteogenesis‐related markers in OPLL cells with GLI1 knockdown after BMP2 treatment for different durations, assessed by WB. (n) Protein expression of osteogenesis‐related markers in PLL cells with GLI1 overexpression after Noggin addition, assessed by WB. (o) ALP and ARS staining in OPLL cells with GLI1 knockdown after BMP2 addition. (p) ALP and ARS staining in PLL cells with GLI1 overexpression after Noggin addition. (q) WB of proteins precipitated using anti‐GLI1 and anti‐BMP2 antibodies. *n* = 3 per group. Data presented as mean ± SEM. Significant levels are *p < 0.05, **p < 0.01, ***p < 0.001 and ****p < 0.0001. ns: No significance. Scale bar: As depicted in the figure.

To gain deeper insights into the regulatory mechanisms of Hh signalling and GLI1 in OPLL, we conducted a PPI network analysis based on transcriptomic data. GLI1, identified as a pivotal hub gene, serves as a central node revealing its close associations with BMP, WNT and NOTCH signalling pathways in our study (Figure 6a and Figure S7). Given the pivotal roles of the BMP pathway in bone formation, we delved further into their interactions with GLI1 in the context of OPLL.

Initially, a marked distinction in the protein expression levels of BMP2 and BMP4 was identified between OPLL and PLL cells (Figure 6b). Next, we observed that knocking down GLI1 in OPLL cells significantly reduced BMP2 and BMP4 protein expression (Figure 6c,e,l). Concurrently, downstream Smad1 showed a decreasing trend, along with a decrease in phosphorylated‐Smad (P‐Smad) 1/5/9 (Figure 6g,l). Conversely, overexpressing GLI1 in PLL cells led to an increase in BMP2 and BMP4 levels, as well as the expression of Smad1 and P‐Smad 1/5/9 (Figure 6c,e,g,i). Furthermore, we noted that antagonising the Hh pathway resulted in reduced expression of BMP2 and BMP4, along with a downregulation of their downstream effectors in OPLL cells (Figure 6d,f,h,k). Activating the Hh pathway in PLL cells similarly exerted an activating effect on the BMP pathway (Figure 6d,f,h,j).

In our quest to deepen our comprehension of the dynamic interplay between the Hh pathway and BMP signalling pathway in OPLL while avoiding off‐target effects, we conducted a series of targeted rescue assays. BMP2 was introduced during the culture of OPLL cells with GLI1 knockdown. We observed that BMP2 introduction could reverse the decreased levels of RUNX2 triggered by GLI1 knockdown, whereas it did not exert the same influence on ALP and OPN (Figure 6m and Figure S8a). Interestingly, the addition of BMP2 also rescued the expression of GLI1 protein following knockdown (Figure 6m and Figure S8a). Consistently, the addition of BMP2 could rescue the decreased levels of ALP secretion and mineralisation induced by GLI1 knockdown (Figure 6o and Figure S8c). In PLL cells overexpressing GLI1, the introduction of the selective BMP antagonist Noggin resulted in the reversal of the upregulation of osteogenesis‐related markers induced by GLI1 overexpression (Figure 6n and Figure S8b). This finding was consistently supported by ALP and ARS staining (Figure 6p and Figure S8d). Furthermore, the introduction of AR‐A014418, an inhibitor of GSK3β, failed to affect the osteogenic capability of PLL and OPLL cells, underscoring the specificity of the Hh–BMP pathway interaction in the osteogenic differentiation of PLL and OPLL cells (Figure S9). Co‐IP further confirmed the interaction between GLI1 and BMP2 (Figure 6q). However, there was no evidence of a comparable binding interaction between GLI1 and BMP4 (Figure S10).

## 4 Discussion

Ossification of the posterior longitudinal ligament (OPLL) is a prevalent degenerative disease in East Asia, with a lack of effective pharmacological treatments and preventive interventions currently available. The uncertain prognosis of surgery, attributed to continuous bone growth leading to recurring and worsening symptoms, underscores the need for a thorough understanding of the key molecules and signalling pathways in OPLL occurrence and development. Current research on OPLL primarily delves into three realms: genetic factors, epigenetic factors and environmental influences [26, 27, 28]. Yet, the specific molecular mechanism remains unclear. Our study uncovered a critical mechanism in OPLL formation—a crosstalk between canonical Hh signalling pathway and BMP signalling pathway, notably highlighted by a positive feedback loop between GLI1 and BMP2.

The current literature indicates that the Hh pathway is implicated in the occurrence of several human diseases, including skeletal disorders. Although recent research has unveiled the involvement of the Hh pathway in osteoblast differentiation and bone formation, whether it acts in an activating or antagonistic manner across diverse temporal and spatial patterns remains unclear. Specifically, activation of the Hh–GLI1 signalling pathway can enhance osteoblastic potential by upregulating osteogenic factors like RUNX2 [29]. However, this paradigm does not extend to dental tissue regeneration, where Shh inhibits stem cell differentiation into bone/dentin [30]. Inconsistent findings persist even in calvarial bone development, where membranous ossification predominates. GLI1^+^ cells, influenced by Ihh from committed osteogenic progenitors, contribute to craniofacial bone turnover and injury repair [31]. However, conflicting perspective suggests that the activation of Hh signalling in mesenchyme for calvarial bone primordia disrupts the osteogenesis development by hindering downstream molecules, such as GLI2 [15]. This raises our profound interest in whether the Hh–GLI1 signalling pathway is involved in the ectopic bone formation of OPLL. To our knowledge, research in this area is still in its infancy, and many questions remain unanswered. Following the identification of GLI1 as a key gene in PLL and OPLL cells through transcriptome sequencing, our functional experiments confirmed its positive role in osteogenic differentiation. It is worth noting that antagonising the Hh pathway reversed the enhanced osteogenic potential resulting from GLI1 overexpression, deriving GLI1's enhancing effect in OPLL back to the canonical Hh pathway.

In a genome‐wide microarray analysis among a Chinese population, Ren et al. identified the BMP4 gene as a contributor for OPLL development and severity [32]. Moreover, a targeted next‐generation sequencing study unveiled a missense mutation in BMP2 associated with OPLL [33]. Despite the comprehensive exploration of the BMP pathway in osteogenesis, research on OPLL remains relatively limited. In the context of cervical OPLL, BMP2 is regarded as a downstream factor affected by the long noncoding RNA XIST [34]. BMP4 has been recently identified as a shared core biomarker in the development of both OPLL and ossification of the ligamentum flavum (OLF) through comprehensive bioinformatics analysis across five GEO databases [35]. Our study echoed these findings, confirming BMP2 and BMP4's involvement in the ossification process of OPLL on protein levels. Furthermore, our transcriptome analysis revealed differential expression patterns of BMP2 and BMP4 in OPLL. PPI network analysis indicated potential connections with GLI1, which was subsequently verified in follow‐up experiments. The Hh and BMP pathways play vital roles in progressive osseous heteroplasia (POH), characterised by membranous ossification and fibrodysplasia ossificans progressiva (FOP), where endochondral ossification is the primary pathological feature respectively. Here, we pointed out for the first time the interaction between the Hh pathway and the BMP pathway in OPLL.

Evidence from various species or tissues indicates that Shh serves as a crucial factor in activating BMP2 in vertebrate limbs, contributing to the establishment of the NKX3.2/Sox9 loop maintained by BMP signalling to induce chondrogenesis in mesenchymal cells [36]. Moreover, GLI2 facilitates Shh‐induced osteogenic differentiation in C3H/10 T1/2 by interacting with the BMP2 promoter [37]. BMP and Hh signalling co‐ordinately regulate murine chondrocyte proliferation, with BMP exhibiting the capacity to upregulate Ihh expression in the absence of the Ihh/PTHrP pathway [38]. In a murine model of injury‐induced ectopic ossification, the initiation of the pathological osteogenic cascade is likely triggered by the establishment of the MSC niche, wherein BMP and Hh signalling are coregulated through feedback mechanisms [39]. Another evidence for human dental pulp stem cells points to the possible involvement of GLI1 and BMP2/BMP4 in regulating the biological characteristics of mature and immature permanent teeth using Shh as a mediator [40]. Nevertheless, the BMP pathway's role in maintaining the dynamic equilibrium between osteoblasts promoting bone formation and osteoclasts promoting bone resorption is invariable. Direct stimulation of osteoclast differentiation by BMP2 and enhancement of its resorptive activity by BMP4 are observed [41]. Clinical evidence supports BMP2 inducing vertebral endplate resorption posttransforaminal lumbar interbody fusion by increasing local inflammatory reactions [42]. In addition, mice overexpressing BMP4 showed a reduction in bone mass due to an increased number of osteoclasts [43]. This may provide insight into the decreased mRNA and protein levels of BMP2 observed in OPLL in this study. Due to the dynamic processes of bone formation and resorption, maintaining a delicate balance between the two is crucial for bone homeostasis. The intricate relationship between the Hh–GLI1 and BMP signalling pathways in this complex system requires further investigation.

Notably, in this study, we comprehensively characterised primary PLL and OPLL cells, revealing clear regularities in their osteogenic differentiation, as evidenced by Western blot analysis of osteogenesis‐related markers. RUNX2 and OPN exhibit distinct expression patterns during osteogenic induction in the two cell types. OPN expression in OPLL cells peaked on day 7, suggesting that these cells are in the early stages of bone matrix development and mineralisation [44]. In contrast, the delayed peak expression of OPN in PLL cells may imply that OPLL cells exhibit specific biological features of osteogenesis earlier than PLL cells, or are closer to the final stages of ossification. Regarding RUNX2 expression, we observed its typical pattern during osteoblast differentiation: weak expression in uncommitted mesenchymal cells, upregulation in preosteoblasts, peaking in immature osteoblasts and downregulation in mature osteoblasts [45]. The abnormal expression pattern of RUNX2 observed in OPLL cells does not contradict the general differentiation process of osteoblasts but rather suggests additional regulatory mechanisms in OPLL that maintain elevated levels of RUNX2 expression.

This paper has certain limitations. Firstly, anterior cervical discectomy/corpectomy and fusion (ACDF/ACCF), commonly employed for OPLL, offer direct access to ossified ligament tissue but provide a limited quantity of tissue, restricting the sample size for our study. Additionally, small sample size and limited tissue availability prevented us from conducting a thorough analysis of OPLL based on radiographic classification (segmental, continuous, circumscribed, mixed type). Finally, despite providing valuable insights at the cellular level, the findings of this study underscore the necessity for broader in vivo investigations using robust models that accurately represent human OPLL pathology in the future.

In conclusion, our current study confirms the crosstalk between the canonical Hh pathway and the BMP pathway in OPLL. Canonical Hh pathway regulates osteogenic differentiation of human cervical PLL cells by BMP signalling pathway, implying that antagonising the Hh pathway or GLI1 could be a promising therapeutic approach for OPLL in the future.

## Author Contributions

**Wenbo Xu:** conceptualization (equal), formal analysis (equal), investigation (equal), methodology (equal), software (equal), visualization (equal). **Bingbing Ran:** conceptualization (supporting), formal analysis (equal), investigation (equal), methodology (supporting), software (equal), visualization (equal). **Toshimi Aizawa:** supervision (equal), validation (equal), writing – review and editing (equal). **Wanguo Liu:** formal analysis (supporting), methodology (supporting). **Jianhui Zhao:** formal analysis (supporting), methodology (supporting). **Renrui Niu:** investigation (supporting), visualization (supporting). **Zeping Liu:** investigation (supporting). **Rui Gu:** conceptualization (equal), funding acquisition (lead), supervision (equal), validation (equal), writing – review and editing (equal).

## Ethics Statement

The study was conducted in accordance with the Declaration of Helsinki, and performed under the guidelines of the institutional review board and the ethics committee of Jilin University (approval No. SY2023072606).

## Consent

All specimens were obtained and utilised in compliance with patient informed consent.

## Conflicts of Interest

The authors declare no conflicts of interest.

## Supporting information


**Figure S1.** Phenotypic features of PLL and OPLL Cells.
**Figure S2**. Associated GO Terms with Upregulated Genes.
**Figure S3**. Bubble Plots Depicting the Enrichment of The Top 20 Upregulated and Downregulated Genes According to WikiPathways.
**Figure S4**. Observation of Cell Transfection Under Fluorescence Microscopy.
**Figure S5**. The Determination of Safe Usage Concentrations for Purmorphamine and Cyclopamine Based on CCK8.
**Figure S6**. Quantification of GLI1 and osteogenic‐related genes protein expression in PLL cells under three conditions: GLI1 overexpression, CPN addition, and a combination of GLI1 overexpression with CPN supplementation.
**Figure S7**. The Complete View of Protein‐Protein Interaction (PPI) Network Analysis.
**Figure S8**. Quantification of GLI1, BMP2, and osteogenic‐related genes protein expression in OPLL cells under three conditions: control, sh‐GLI1, and BMP2 addition following GLI1 knockout at various time points (0–240 min).
**Figure S9**. Evaluation of RUNX2 protein expression in PLL and OPLL through western blot analysis under four conditions: control, PM introduction, AR‐A014418 introduction and PM+AR‐A014418 introduction, with statistical analysis.
**Figure S10**. Lack of Interaction Between Gli1 and Bmp4.

**Table S1.** Patient demographics and clinical details for sample collection.
**Table S2**. Primer sequences used in this study.
**Table S3**. Reagents used in the study.

## Data Availability

The data that support the findings of this study are available from the corresponding author upon reasonable request.
