# Supplementary material for: The Hedgehog–GLI1 Pathway Regulates Osteogenic Differentiation of Human Cervical Posterior Longitudinal Ligament Cells by BMP Signalling Pathway
Source: J Cell Mol Med. 2025 Feb 5;29(3):e70393. doi: 10.1111/jcmm.70393 (PMC11798735; doi:10.1111/jcmm.70393)
Supplement: Supplementary file 1 — Figure S1. Phenotypic features of PLL and OPLL Cells. Figure S2. Associated GO Terms with Upregulated Genes. Figure S3. Bubble Plots Depicting the Enrichment of The Top 20 Upregulated and Downregulated Genes According to WikiPathways. Figure S4. Observation of Cell Transfection Under Fluorescence Microscopy. Figure S5. The Determination of Safe Usage Concentrations for Purmorphamine and Cyclopamine Based on CCK8. Figure S6. Quantification of GLI1 and osteogenic‐related genes protein expression in PLL cells under three conditions: GLI1 overexpression, CPN addition, and a combination of GLI1 overexpression with CPN supplementation. Figure S7. The Complete View of Protein‐Protein Interaction (PPI) Network Analysis. Figure S8. Quantification of GLI1, BMP2, and osteogenic‐related genes protein expression in OPLL cells under three conditions: control, sh‐GLI1, and BMP2 addition following GLI1 knockout at various time points (0–240 min). Figure S9. Evaluation of RUNX2 protein expression in PLL and OPLL through western blot analysis under four conditions: control, PM introduction, AR‐A014418 introduction and PM+AR‐A014418 introduction, with statistical analysis. Figure S10. Lack of Interaction Between Gli1 and Bmp4. [file JCMM-29-e70393-s002.docx]

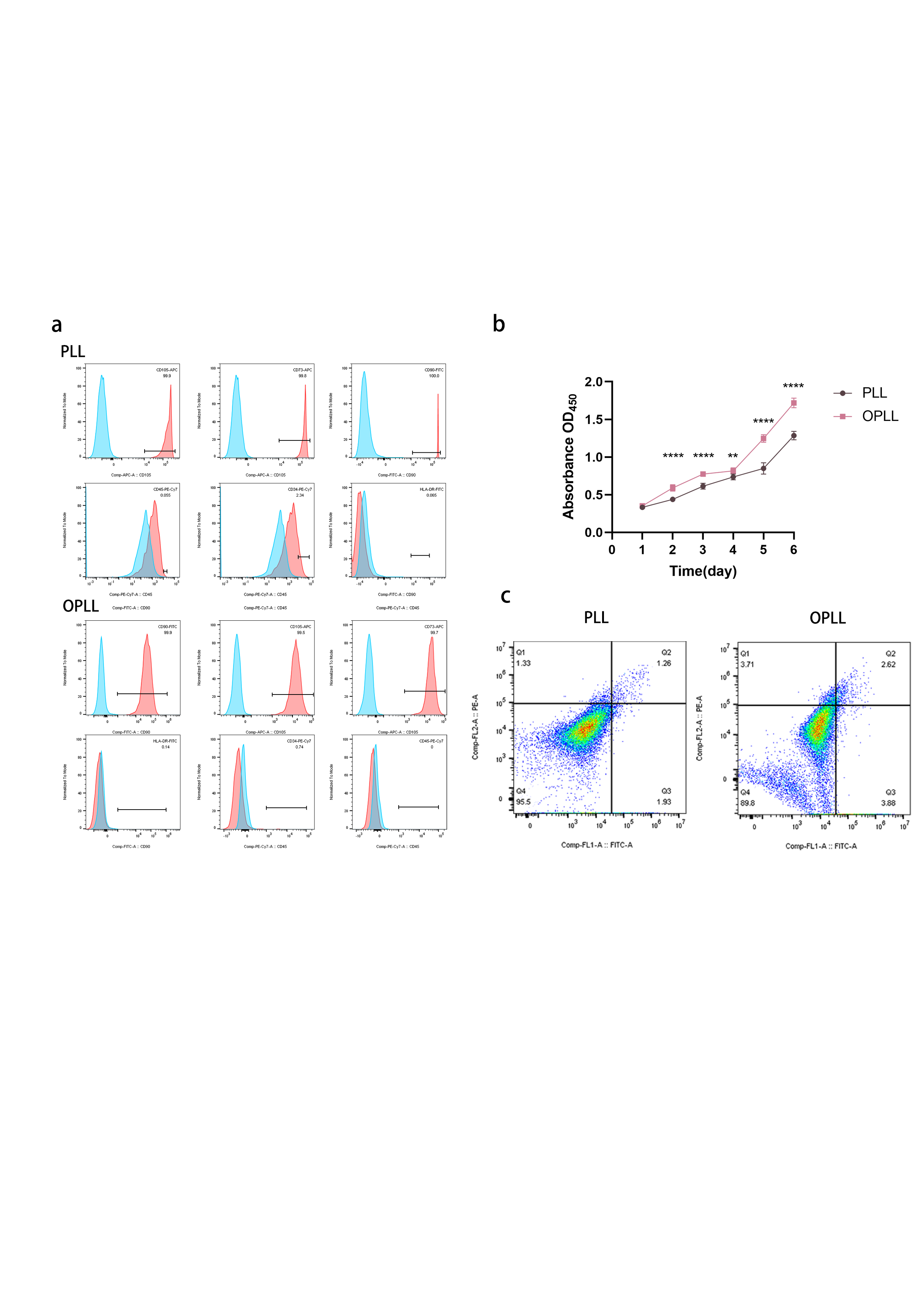


Supplementary Figure S1. Phenotypic features of PLL and OPLL Cells.

(a) The expression of typical MSC surface markers, CD105, CD73, and CD90 were positive in both PLL and OPLL cells, while CD45, CD34, and HLA-DR were negative. (b) Evaluation of cell proliferation capacity in PLL and OPLL cells from day 1 to day 6 using the CCK-8. (c) Evaluation of apoptotic features in PLL and OPLL cells using flow cytometry.


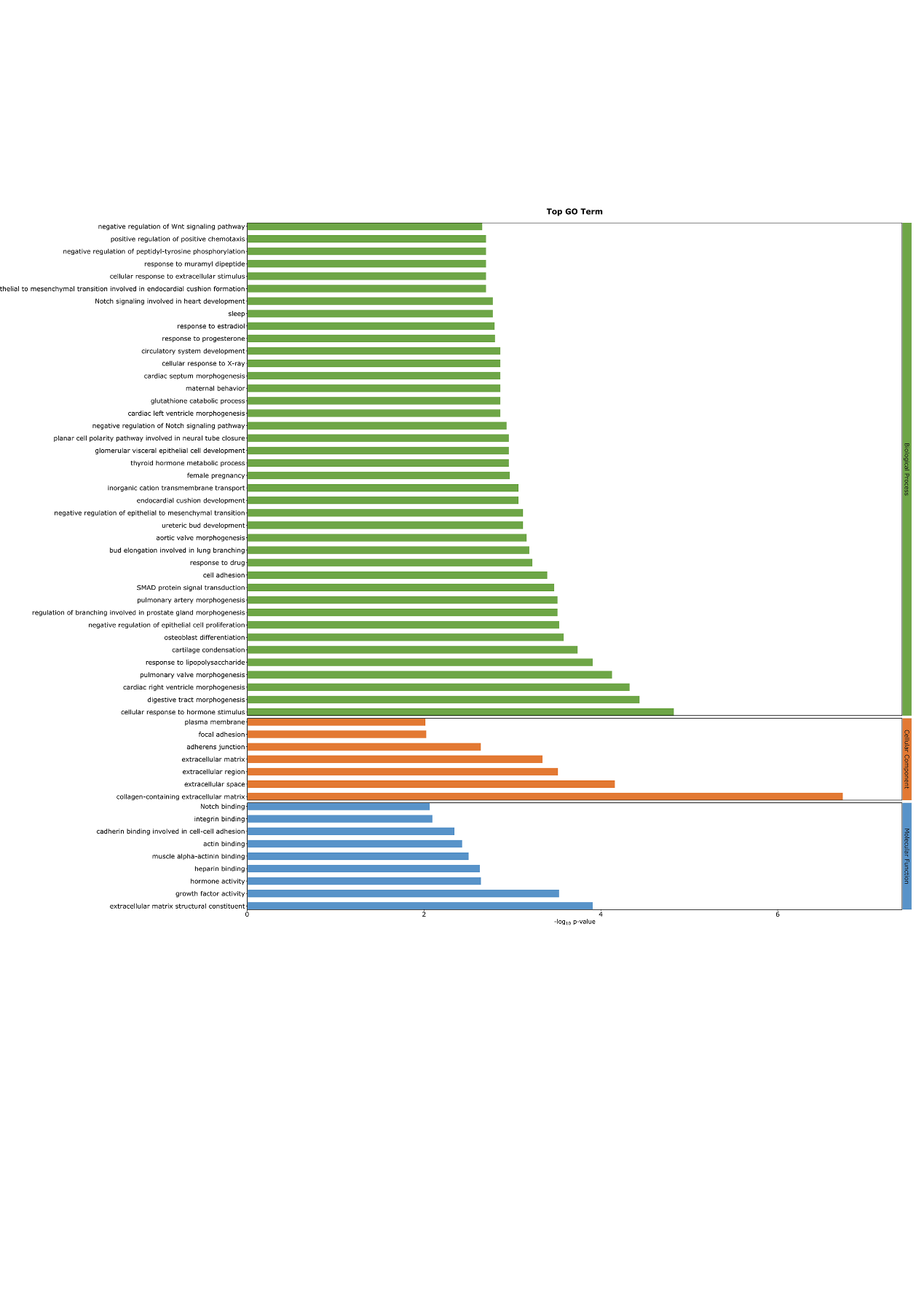


Supplementary Figure S2. Associated GO Terms with Upregulated Genes.


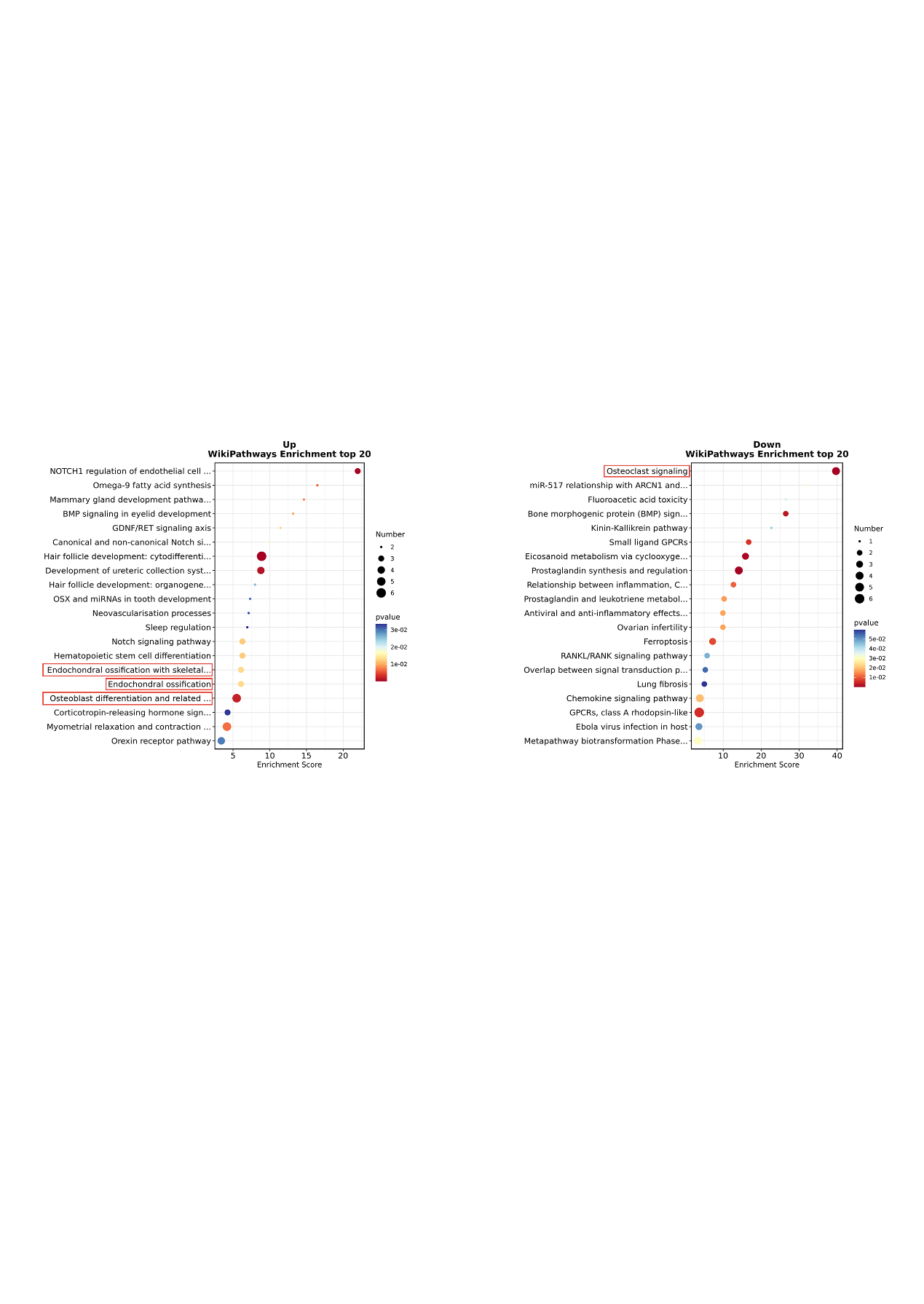


Supplementary Figure S3. Bubble Plots Depicting the Enrichment of The Top 20 Upregulated and Downregulated Genes According to WikiPathways


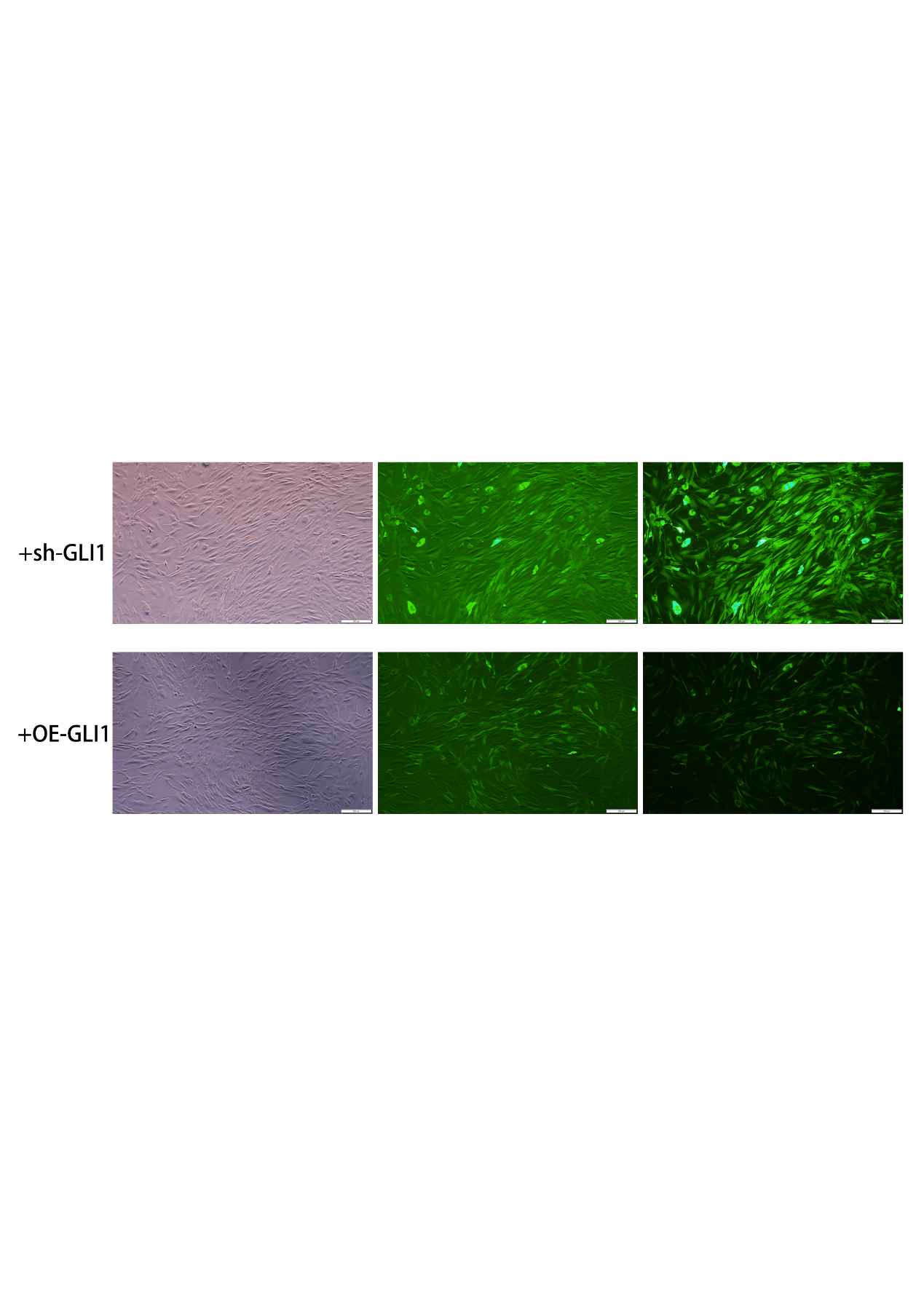


Supplementary Figure S4. Observation of Cell Transfection Under Fluorescence Microscopy.


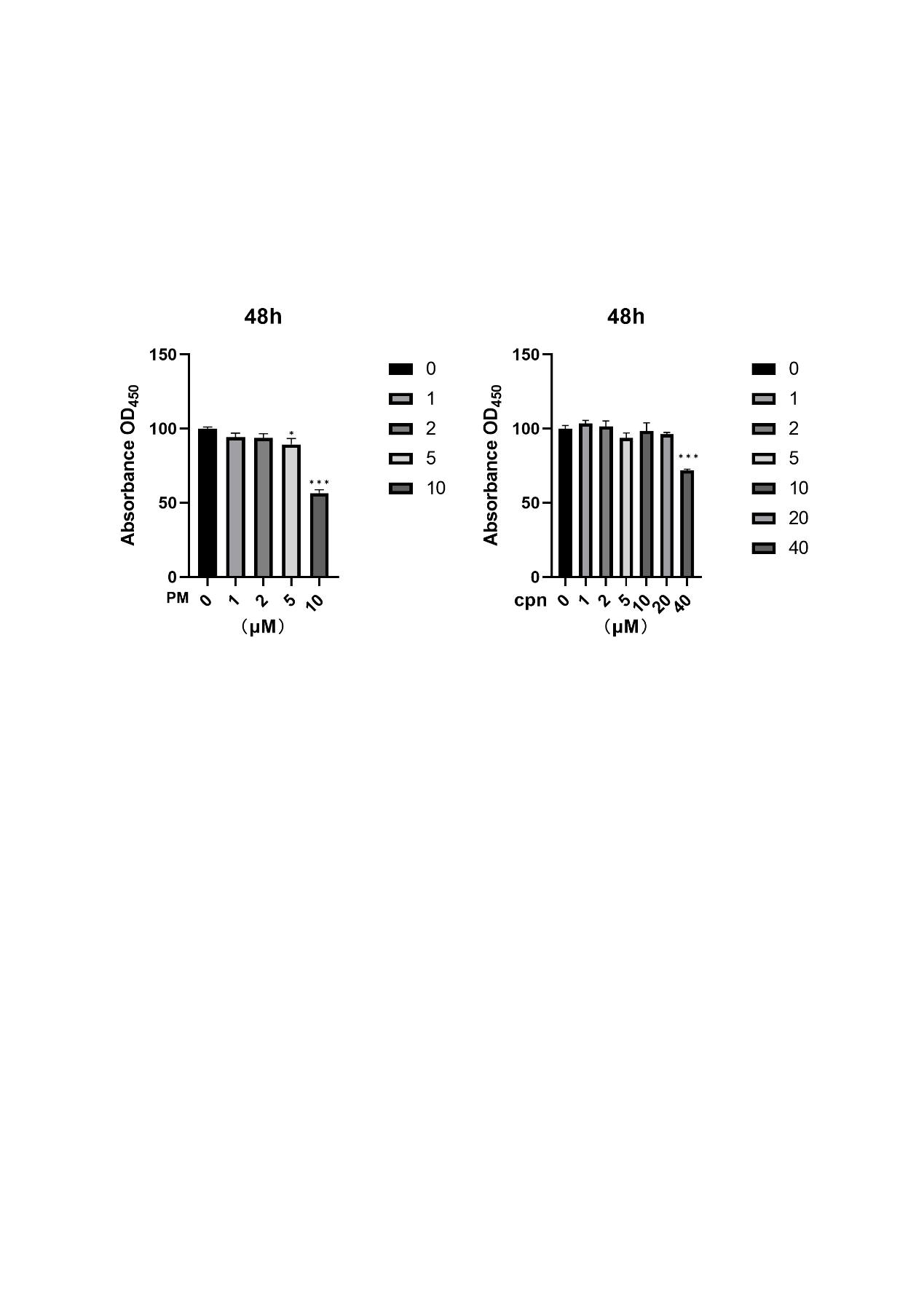


Supplementary Figure S5. The Determination of Safe Usage Concentrations for Purmorphamine and Cyclopamine Based on CCK8. Data presented as mean ± SEM. Significant levels are * p＜0.05, **p＜ 0.01 and ***p＜0.001.


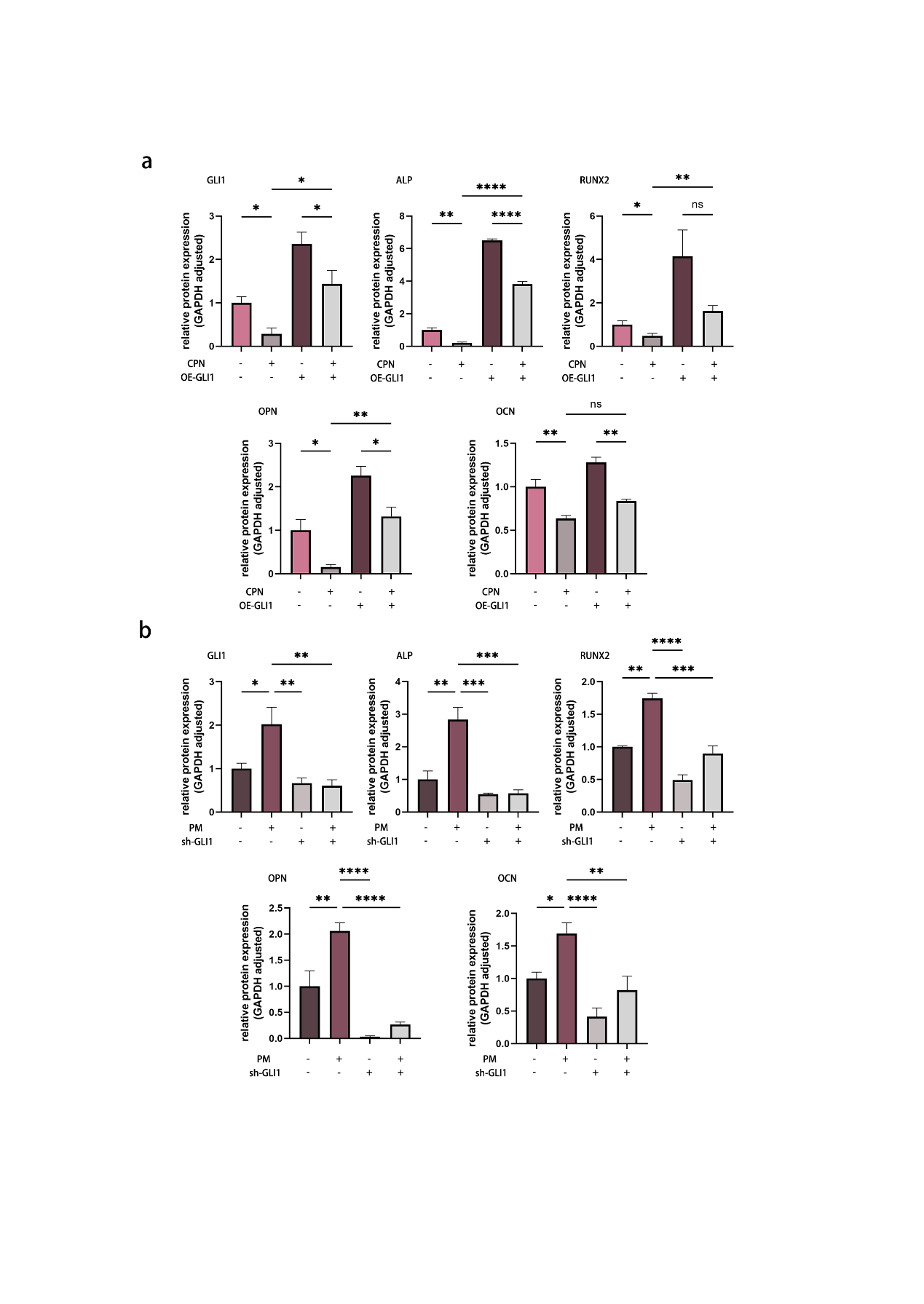


Supplementary Figure S6. (a) Quantification of GLI1 and osteogenic-related genes protein expression in PLL cells under three conditions: GLI1 overexpression, CPN addition, and a combination of GLI1 overexpression with CPN supplementation. (b) Quantification of GLI1 and osteogenic-related genes protein expression in OPLL cells under three conditions: GLI1 knockdown, PM addition, and a combination of GLI1 knockdown with PM supplementation. Data presented as mean ± SEM. Significant levels are * p＜0.05, **p＜ 0.01, ***p＜0.001 and ****p＜0.0001.


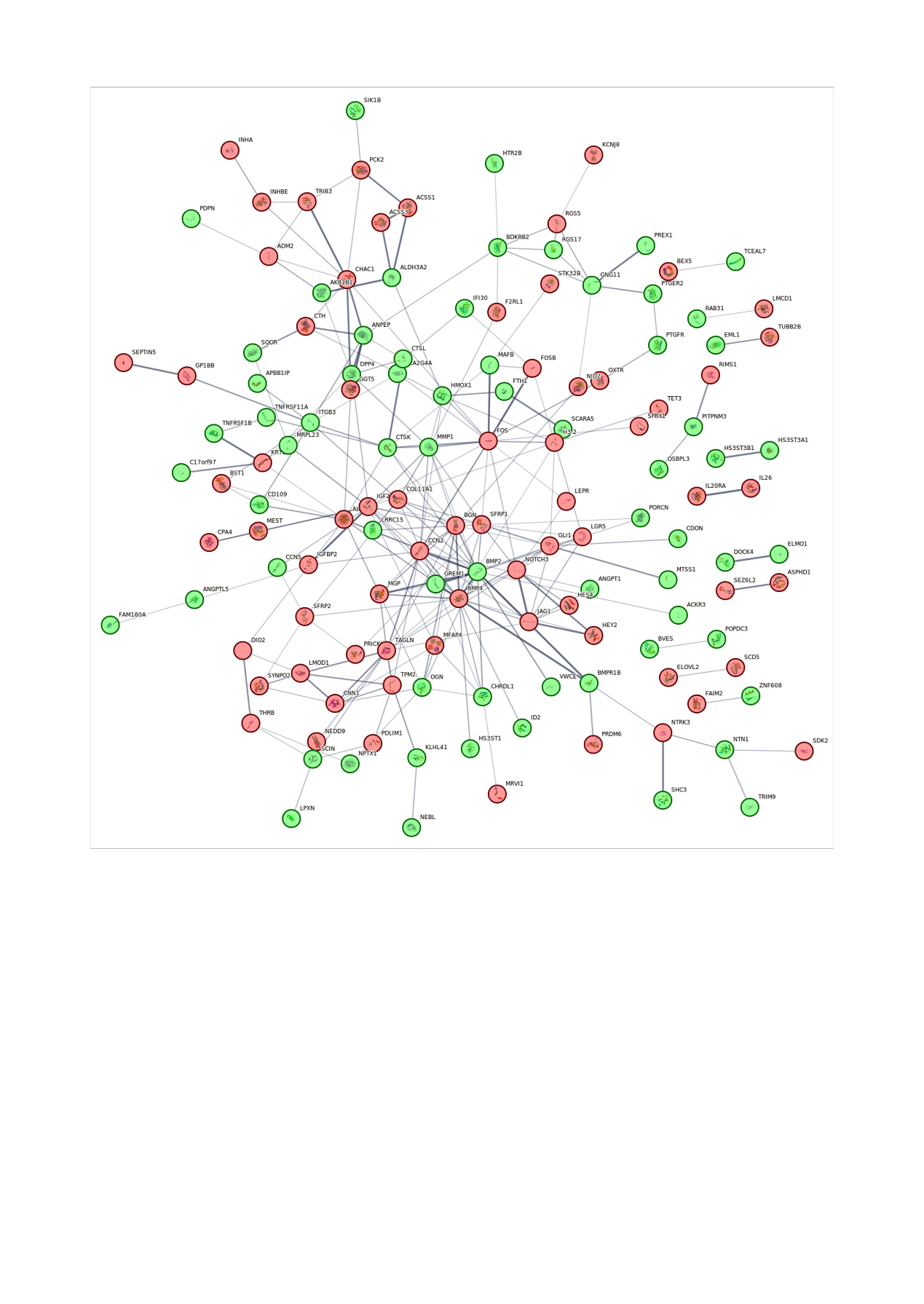


Supplementary Figure S7. The Complete View of Protein-Protein Interaction (PPI) Network Analysis.


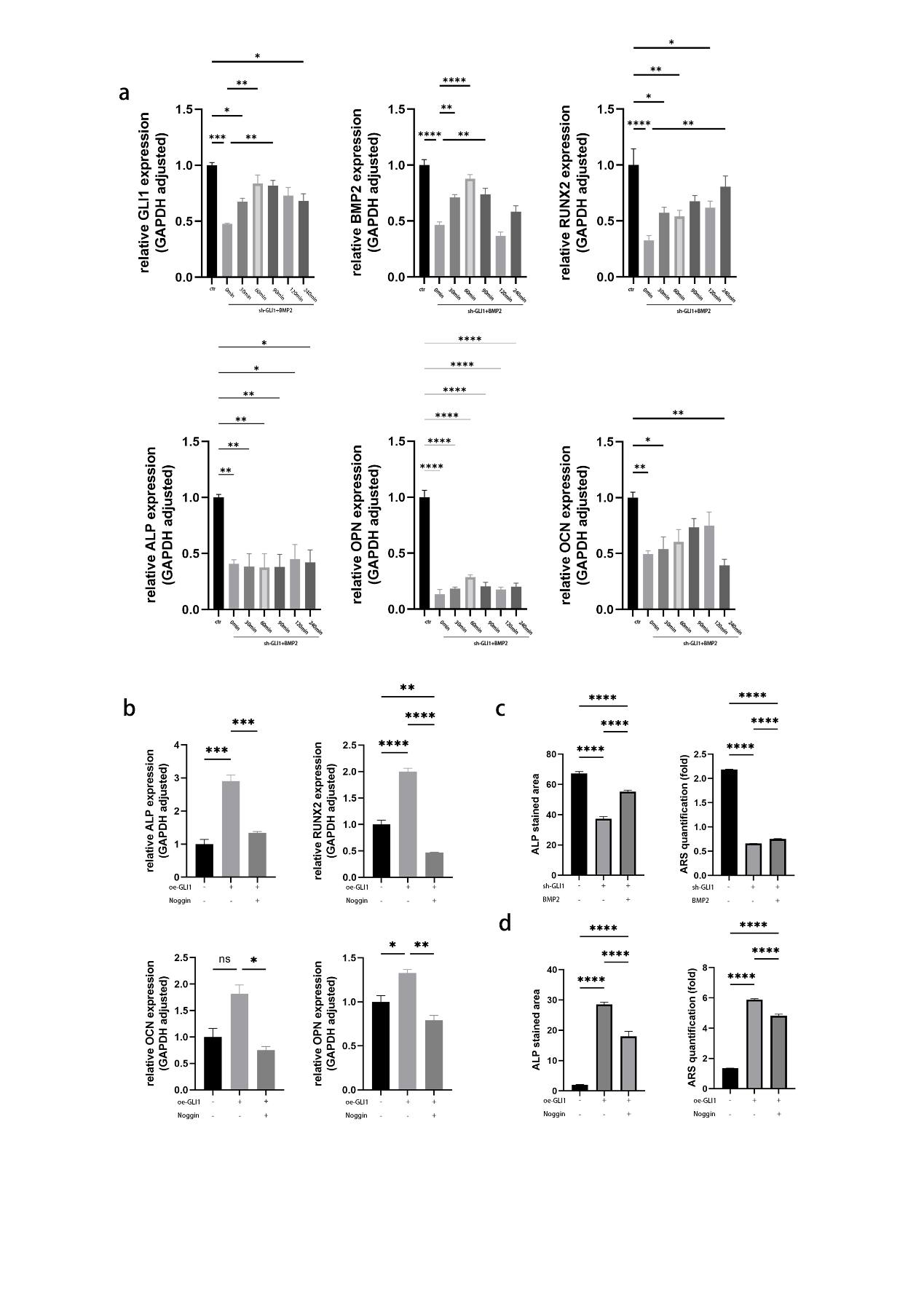


Supplementary Figure S8. (a) Quantification of GLI1, BMP2, and osteogenic-related genes protein expression in OPLL cells under three conditions: control, sh-GLI1, and BMP2 addition following GLI1 knockout at various time points (0-240 minutes). (b) Quantification of osteogenic-related genes protein expression in PLL cells under three conditions: control, oe-GLI1, and Noggin addition following GLI1 overexpression. (c) Quantification of ALP and ARS staining in OPLL cells under three conditions: control, sh-GLI1, and BMP2 addition following GLI1 knockout at 48h. (d) Quantification of ALP and ARS staining in PLL cells under three conditions: control, oe-GLI1, and Noggin addition following GLI1 overexpression at 48h. Data presented as mean ± SEM. Significant levels are * p＜0.05, **p＜ 0.01, ***p＜0.001 and ****p＜0.0001. ns: no significance.


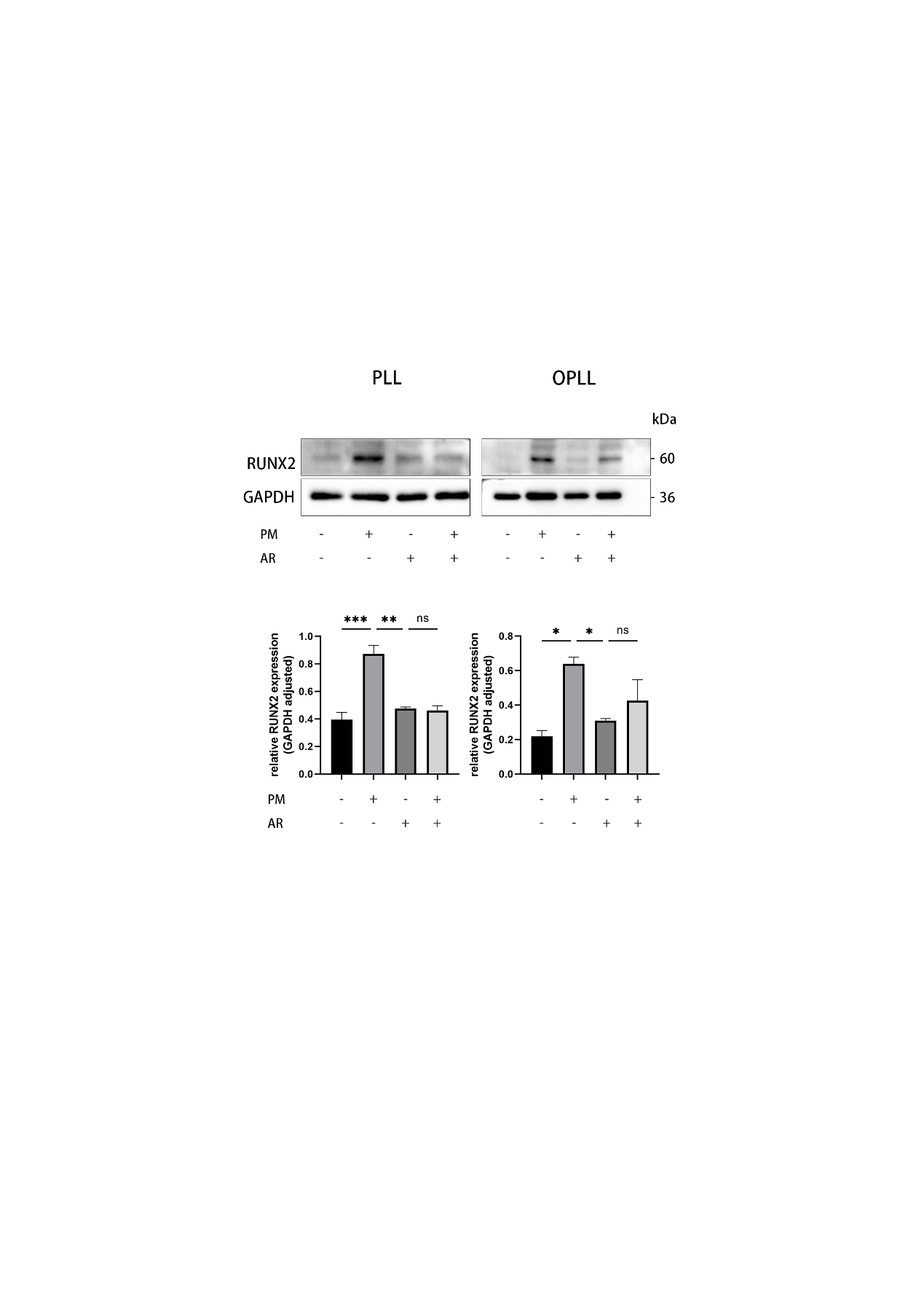


Supplementary Figure S9. Evaluation of RUNX2 protein expression in PLL and OPLL through western blot analysis under four conditions: control, PM introduction, AR-A014418 introduction and PM+AR-A014418 introduction, with statistical analysis. Data presented as mean ± SEM. Significant levels are * p＜0.05, **p＜ 0.01, ***p＜0.001 and ****p＜0.0001. ns: no significance.
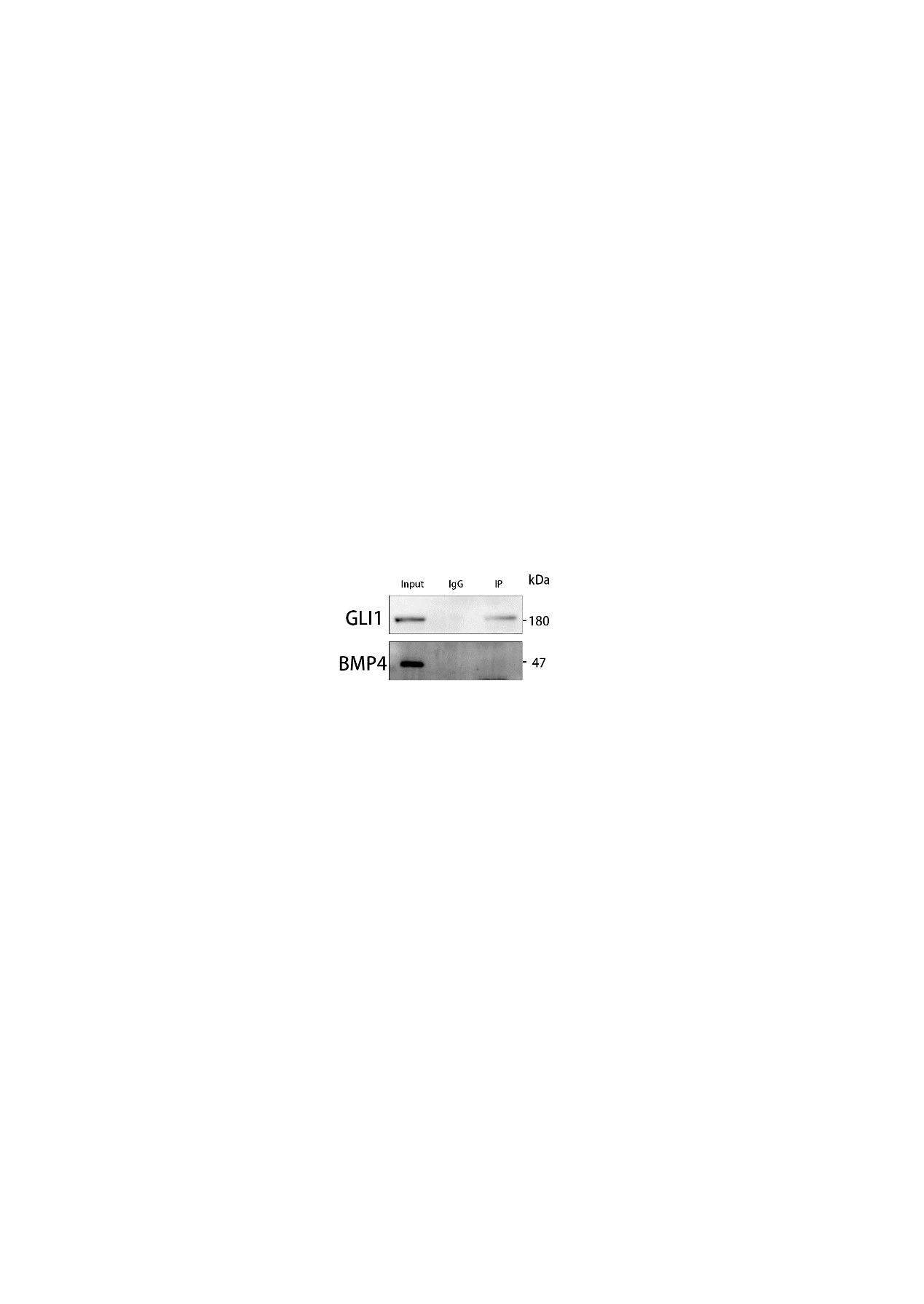


Supplementary Figure S10. Lack of Interaction Between Gli1 and Bmp4.
